# Research needs of higher specialist trainees in psychiatry in Ireland: mixed methods study

**DOI:** 10.1192/bjb.2024.91

**Published:** 2025-10

**Authors:** Eimear Counihan, Cornelia Carey, Anna Feeney, Kevin Lally, Ciara O'Connor, Anne M. Doherty

**Affiliations:** 1 National Forensic Mental Health Services, Dublin, Ireland; 2 Cluain Mhuire Mental Health Service, Blackrock, Dublin, Ireland; 3 St Vincent's Hospital Fairview, Dublin, Ireland; 4 National Drug Treatment Centre, Dublin, Ireland; 5 St Patrick's University Hospital, Dublin, Ireland; 6 Mater Misericordiae University Hospital, Dublin, Ireland; 7 University College Dublin, Dublin, Ireland

**Keywords:** Qualitative research, education and training, curriculum, trainee research, academic psychiatry

## Abstract

**Aims and method:**

Higher specialist trainees (HSTs) in psychiatry in Ireland were recruited to complete a 21-item online questionnaire anonymously. Questions were designed to establish the research experience of HSTs in various years of training, identify perceived barriers to participation and generate potential strategies to overcome these barriers.

**Results:**

Of 165 HSTs surveyed, 50 (30%) responded. Most respondents (58%) were in the second or third year of HST. Most (72%) were training in general adult psychiatry. Themes that emerged from analysis of the qualitative data were ‘collaborative research culture’, ‘guidance’, ‘choice’ and ‘access to resources’. Participants felt they needed more structured guidance and regular supervision, and expressed a desire for more networking and collaboration.

**Clinical implications:**

The need for a supportive, collaborative research culture within psychiatry was predominant among responses. Structured research programmes and access to resources may facilitate a more positive research culture and should be considered as part of the training curriculum.

Engaging in critical analysis of emerging research is a key component of evidence-based medicine and clinical medical education.^1^ The need for research literacy is pertinent in light of the proliferation of highly productive researchers worldwide in the field of clinical medicine in recent years.^2^ With this increased output clinicians must be equipped to discern high-quality publications and there is increasing interest in standardising benchmarks for evidence-based practice.^3^ Research participation is also expected as part of postgraduate medical training globally. However, the support in place to facilitate this varies widely,^4^ with some positive results from mentoring programmes.^5^ Although journal clubs and postgraduate examinations are useful in promoting research literacy,^6^ research participation is central to developing a full understanding of the research process and literature. Participation in research has been shown to improve outcomes in patient care.^7,8^ Participation should begin as early as possible, ideally from an undergraduate level and at least prior to completion of training.^9^

A number of recent studies have investigated barriers and facilitators to research participation in training.^10–12^ Studies have identified a lack of protected time as a barrier to research. Lack of familiarity with research methods, lack of funding, lack of access to databases and lack of supervision have also been cited.^10^ In a study carried out among clinicians in Ireland, career progression was the strongest factor motivating participation in research.^11^ Other strong motivators include having a supportive supervisor and interest in a topic. In a study of paediatric residency programmes in the USA,^4^ characteristics associated with high-participation programmes included a scholarly activity requirement, mentorship by >25% of faculty and programme director belief that all residents should present work regionally or nationally. Mentoring and teaching are mandatory aspects of postgraduate training curriculums. Despite the important role of mentorship and evidence of its benefits,^13^ there is no standard definition in the literature, with meanings of the term ‘mentorship’ ranging from coaching and advising to advocacy and role modelling.^14–16^

The College of Psychiatrists of Ireland offers a dedicated research day each week as part of higher specialist training (HST). The provision of a research day marks out the importance of research and evidence-based medicine within the psychiatry training curriculum. However, time is only one barrier to research participation. At present, on the psychiatry HST programme the burden of research supervision falls on individual clinical training placements, which change yearly and where the clinical supervisor may not have a research interest. A trainee mentoring service has been established in recent years by the *Irish Journal of Psychological Medicine*, which trainees are invited to access for guidance and mentorship regarding research. However, the parameters of this service are not clearly defined and trainee awareness of this service has not previously been described.

To our knowledge, the experience of psychiatric higher specialist trainees (HSTs) engaging in research has not been previously studied. This study aimed to determine the level of participation in research of HSTs training in psychiatry, to explore the feasibility of mentorship programmes and how best to enhance the trainee research experience.

## Method

### Participants

An 21-item online survey was disseminated to all psychiatric HSTs in Ireland via the training body email database. An information leaflet describing the nature and purpose of the study was included in the email, and participant consent was obtained prior to completion of the survey. All 165 psychiatric HSTs were eligible to participate. The survey was live from October 2023 to December 2023. Of note, the training year in Ireland runs from July to July. To preserve anonymity, participants were not asked for a name or any identifying information, including gender, race/ethnicity or information pertaining to their place of work. Survey data were anonymously downloaded to Microsoft Excel.

### Survey development

The survey was designed with the intention of conducting thematic analysis.^17,18^ Data were collected using surveys rather than interviews, to retain participant anonymity and to allow for a larger sample. The survey was designed by a working group of psychiatric trainees, informed by a literature review. Survey items were designed to establish the experience of HSTs in various years of training, to elicit HSTs’ concerns in relation to their research experience, to identify perceived barriers to participation in research and to generate potential strategies to overcome these barriers. Survey items included closed questions relating to stage of training and training scheme, and open-ended questions relating to experience of research and suggestions for how research participation could be better facilitated. Ethical approval was granted by the Royal College of Physicians of Ireland Research Ethics Committee (RECSAF 195).

### Data analysis

Reflexive thematic analysis was carried out using Braun & Clarke's six-phase guide,^17,18^ involving becoming familiar with the data, generating initial codes, searching for themes, reviewing themes, defining and naming themes and writing up the findings. This flexible inductive approach allowed researchers to consider their own values and opinions and how these might affect the analysis, while also allowing themes to emerge from the participants’ responses, rather than adhering to predetermined themes.

NVIVO analysis software (QSR International) was used to analyse the data. Survey responses were imported into NVIVO and the two primary researchers (E.C. and C.C.) separately carried out initial open coding using the same data. Codes were generated by analysing each free-text survey response. The two sets of open data were then compared and themes were established through discourse. Simple descriptive statistics were used for closed questions.

## Results

A total of 30% of participants (50/165) responded. Demographic details such as gender and nationality were not included, to preserve participant anonymity. Of those who responded, the majority (58%) were in the second or third year of HST. The majority were training in general adult psychiatry (70%) and 57% of those respondents were also training in a subspeciality. Characteristics of respondents are summarised in Table 1. When asked ‘Do you have a plan for your research day?’, 13 respondents (26%) said no. When asked ‘Do you have adequate access to research supervision?’, 18 (36%) said no.

**Table 1:** Key characteristics of respondents (*n* = 50)

	Respondents, *n* (%)
**Year of higher specialist training (HST)**	
Year 1	8 (16%)
Year 2	12 (24%)
Year 3	17 (34%)
Year 4+	13 (26%)
**Specialty**	
Child and adolescent mental health services (CAMHS)	13 (26%)
Dual general adult psychiatry and CAMHS	2 (4%)
General adult psychiatry	35 (70%)
Psychiatry of intellectual disability	7 (14%)
Liaison psychiatry	1 (2%)
Psychiatry of later life	12 (24%)
**Higher degree**	
PhD completed	1 (2%)
PhD commenced	5 (10%)
MD completed	5 (10%)
MD commenced	7 (14%)
Masters completed	12 (24%)
Masters commenced	10 (20%)
**Research experience**	
Involvement in multiple research projects throughout HST	33 (66%)
Publication in print	29 (58%)
Publication in print prior to HST	22 (44%)
Publication online	32 (64%)
Publication online prior to HST	25 (50%)
Poster presentation	45 (90%)
Oral presentation at an academic conference	25 (50%)
**Research supervisor**	
Current educational supervisor	9 (18%)
Previous educational supervisor	2 (4%)
Academic researcher	10 (20%)
Other consultant psychiatrist	9 (18%)
No response	19 (38%)

### Specific obstacles and willingness to receive and provide mentorship

**Fig. 1:**
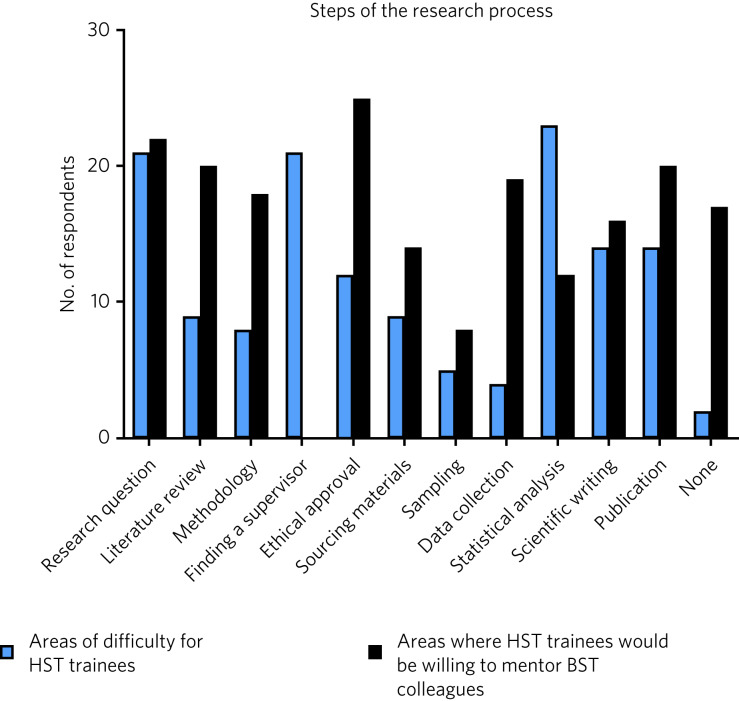
Areas of difficulty in the research process for psychiatry trainees in higher specialist training (HST) and areas in which they would be willing to mentor junior colleagues in basic specialist training (BST).

Of the 50 respondents, 32 (64%) were not aware of the trainee mentoring service, 10 (20%) were ‘vaguely’ aware and 8 (16%) were aware of the service. Were a research mentorship programme available through the professional college body, 40 respondents (80%) said they would avail of it. Respondents endorsed greatest difficulty with forming a research question (42%), finding a supervisor (42%) and statistical analysis (46%). Of all the respondents, 34 (68%) would be willing to mentor a trainee on the basic specialist training (BST) scheme through the research process, with 50% willing to supervise the ethical approval process and 44% willing to supervise the formation of a research question. Areas of difficulty reported by HSTs compared with areas where they would be willing to provide mentorship to junior trainees are presented in Fig. 1.

### Common themes

Four common themes were elicited from the free-text responses: a collaborative research culture, choice, guidance and access to resources. The detailed semantic themes and latent themes are presented in Supplementary Appendix 1, available at https://doi.org/10.1192/bjb.2024.91.

#### Collaborative research culture

Collaborative working and the production of valuable research were strongly endorsed by respondents. Many trainees who had already engaged in research found it to be an isolating experience and several expressed a desire for networking and collaboration. Trainees viewed collaboration as a motivator. Trainees also viewed this as a way to produce better-quality research:

‘Part of research is being able to collaborate and network with the experts, you need to know who to talk to and be proactive in your approach, projects that are imposed on uninterested people tend to have low value’

‘I need someone to motivate me and prompt me’.

Respondents were primarily focused on skills development rather than publications. Many respondents felt that it would be helpful to have more training in research-related skills, for example there were suggestions for ‘teaching sessions on how to best organise and plan for the year’, masterclasses, guidance on applying for ethical approval, and advice and guidance on pursuing a research degree. It was suggested that those in academia who have the requisite skill sets do not have the time to foster a culture of research participation among the trainee group as a whole:

‘Academics who are publishing are time poor and have multiple trainees and/or limited areas of interest and therefore fostering and developing research skills are restrained’.

#### Choice

Participants reported a lack of choice in research projects and supervisors. Some reported difficulty finding any research supervisor and others outlined a situation where they felt obliged to engage in projects with their educational supervisor even though it was not an area of interest. Respondents suggested that the training body could assist in providing trainees and supervisors with greater choice in this area:

‘Educational supervisor has reluctantly agreed to supervise in absence of another consultant’

‘It would be helpful if the college supported some sort of matching of research supervisors and trainees’.

Respondents viewed mandatory research participation as an obstacle to career development. Some expressed concern regarding the neglect of other areas, such as management and psychotherapy, and some associated participation in research with a future career in academia, rather than a part of routine clinical practice:

‘If it helps career development – though don't plan on being involved in academic[s]’.

#### Guidance

The need for appropriate guidance appeared frequently in the text. This pertained to guidance by a supervisor, guidance from the training body and peer-to-peer guidance. Trainees expressed concern that they might not take the correct approach to research and that better guidance would help ‘To ensure I am on the right path’. Trainees cited the need for an ‘experienced’ and ‘enthusiastic’ supervisor:

‘Struggled to find a mentor. Not something that was of interest to a lot of consultant[s] where I am working. Those that were are very selective of who they help with research’

‘Now that I have a research supervisor I feel that I am learning and proceeding with research; earlier in my career it would have been helpful to have more guidance around getting involved in research and planning projects’.

Trainees suggested that the training body could assist in structuring and planning research participation throughout the higher training scheme. Some respondents spoke about the planning that goes into a substantial research project; others wanted guidance on how to plan and appropriately use their research day:

‘Clearer guidance in early stages of HST re building research question, progressing ethics and accessing research supervisor – ideally within first few weeks of commencing HST each July, to then use as guidance for rest of training. At later stages of training this could focus on academic writing and getting published, or building grant applications etc.’

‘A better system to allow trainees to engage in research of interest across HST rather than ad hoc projects’.

Trainees were not only interested in guidance by senior colleagues and acknowledged that this may not always be achievable, given time constraints – peer-to-peer guidance was also endorsed:

‘Advice from people who have done similar research to myself, at a peer to peer level’.

However, although many respondents were interested in providing supervision and/or mentorship to junior colleagues, a perceived lack of competencies was reported as a barrier:

‘I do not feel I would have enough experience or knowledge to supervise a BST [basic specialist training] trainee’.

#### Access to resources

Respondents reported that a lack of resources was impinging on their ability to complete research. This included a lack of time, with the research day not always constituting protected time: some participants stated that ‘clinical responsibilities can bleed into this time’ and cited difficulties with ‘boundaries between clinical and non-clinical work’. Completing other learning outcomes as outlined by the training body was also reported as a limiting factor:

‘The grid. There's no other time to do all the outcomes on it’.

Respondents cited financial barriers to research participation, particularly in relation to postgraduate degrees:

‘Postgrad education, such as MD/PhD, might not be financially feasible for some, even with TSS [Training Support Scheme] grant etc.’.

Geographical inequalities were also cited as an issue, with trainees in more remote regions having less access to research opportunities. A central hub was suggested as a way to partially compensate for this inequality:

‘It would be interesting if there were a central hub (like the jobs advertised on the college's website) where consultant researchers could list projects they need help with, and trainees could contact them’.

Respondents suggested specific practical resources that would be helpful, such as access to training courses in statistics and statistical packages, access to a librarian, access to a statistician and free access to journals.

## Discussion

Most survey respondents were training in general adult psychiatry and there was an even spread of respondents across the years of higher specialist training (HST). Just over a quarter of respondents had no plan for their research day and over a third reported having inadequate access to research supervision. Just 22% of respondents reported engaging in research with their current or previous educational supervisor. A substantial number of HST trainees responding to this survey did not feel comfortable in supervising a junior colleague in basic specialist training (BST) with research. As some of these HST trainees are approaching the end of HST and will soon be working as consultants with trainees under their clinical supervision, this presents a significant gap in training that perpetuates a cycle.

As a group, forming a research question, finding a supervisor and statistical analysis were the areas where most respondents got ‘stuck’ with research. However, 22 endorsed mentoring individuals in BST in forming a research question, with 8 of these being the same individuals who endorsed feeling ‘stuck’ with forming a research question themselves. This may indicate a large gap between those respondents who become stuck in forming a research question and those who would feel comfortable mentoring a junior colleague in doing the same. Given that eight respondents would supervise junior colleagues despite having difficulties forming their own research question it may be the case that some respondents have a higher expectation of the quality of their own research than that of junior colleagues. Surprisingly, publication was an area of lesser concern and 40% (*n* = 20) of respondents would feel comfortable supervising a BST through the publication process.

Contrary to what was expected and what has been cited in the literature,^11^ respondents focused on research culture and skills development rather than the role of research in career development. In fact, some respondents viewed research time as a barrier to other aspects of career development, such as training in management and psychotherapy. This contrasts with findings that publication rate and clinical performance actually correlate positively with one another.^19^ There was a sense that some respondents were seeking an external source of motivation, as well as guidance. This may be linked to the sense of isolation some trainees described, and may be alleviated by promoting a culture of collaboration and support for HSTs involved in research.

Although some respondents felt that choice in which projects they were involved in and who supervised them was important, there were some who reported a desire to be allocated a supervisor for the duration of their HST. This may be due to the fact that there is no clear pathway for engaging in research throughout HST. Many trainees found it difficult to approach academic researchers without having a prior connection. Some trainees find their ability to link with a supervisor with appropriate experience hampered by their geographical location. Availability of online supervision, and online masterclasses and skills training, may alleviate the difficulties associated with a rural placement, for example.

Factors that have been shown to impede clinician participation in research include lack of protected time, lack of skills and lack of supervision. The lack of protected time has been ameliorated through the provision of a protected research day at HST level, but some trainees still have clinical obligations that carry over into this time. Trainees responding to this survey also cited lack of access to resources as a barrier. Although a limited HST fund can be utilised for statistical packages and other educational tools, more straightforward provision of these resources at an organisational level, as is the case with universities, may improve the accessibility of such resources. In addition to the difficulty accessing these resources, trainees may also lack confidence in using them without formal training being available.

### Strengths and limitations

One strength of this study was the recruitment from a national sample of HSTs, including all subspecialty groups. There were a number of limitations to this study. Participant recruitment was a challenge. The response rate (50 respondents of 165 invitations to participate) overall was 30%. However, this is consistent with response rates to online surveys by doctors from various medical specialties, which in one study averaged 35%.^20^ There might be a risk of selection bias, whereby those most interested in research at HST level or those most frustrated by their experience of research, might have participated in the study. Furthermore, 6 (12%) had completed a PhD/MD and a further 24% (12) had commenced a PhD/MD, which may indicate a bias towards those who are more active in research; however, a broad range of respondents, with a broad range of experience, were captured. The primary researchers in this study of psychiatry non-consultant hospital doctors (NCHDs) were psychiatry NCHDs themselves. This increased the risk that conclusions would reflect their own experiences, although bracketing was used to attenuate this.^21^ We did not collect data on gender or race/ethnicity, which are recognised barriers to research participation.^22–26^ Lastly, although lack of access to research in more rural areas was mentioned in the free-text responses, we did not collect quantitative data pertaining to this.

### Practical implications and future research

The results of this survey support the introduction of a number of initiatives. HST trainees in psychiatry endorsed the need for mentorship programmes and the majority were willing to mentor junior colleagues. As in previous studies, trainees cited a lack of skills as impeding research and mentoring ability. The College of Psychiatrists of Ireland (CPsychI) provides HST masterclasses; more masterclasses focusing on research-related skills may be beneficial. A research mentorship programme for new consultants enabling them to supervise junior trainees’ research with confidence may also be merited. The CPsychI has also recently established a ‘Research Hub’ accessible online to trainees via its website. Currently this hub includes research FAQs and a notice board for those with projects seeking to recruit interested trainees. It will be important to evaluate these initiatives over time to ensure that they are meeting training needs.

Future research in this area should include a further survey of HSTs. This should include collection of data on gender, ethnicity and whether trainees are based in a rural or an urban setting, as these have been identified as significant barriers to participation in research. Strategies to increase participation in future surveys could be utilised, for example by sending follow-up email reminders to complete survey questionnaires.^27^ In addition, there is a need for qualitative research to explore the experience of research for HSTs in greater depth and to analyse the enablers and barriers to research participation.

## Supporting information

Counihan et al. supplementary material 1 Counihan et al. supplementary material

Counihan et al. supplementary material 2 Counihan et al. supplementary material

## Data Availability

The data that support the findings of this study are available from the corresponding author, E.C., upon reasonable request.

## About the authors

**Eimear Counihan** is a senior registrar in forensic psychiatry with the National Forensic Mental Health Services, Dublin, Ireland. **Cornelia Carey** is a senior registrar in general adult psychiatry with Cluain Mhuire Mental Health Service, Blackrock, Dublin, Ireland. **Anna Feeney** is a senior registrar in general adult psychiatry at St Vincent's Hospital Fairview, Dublin, Ireland. **Kevin Lally** is a senior registrar in addiction psychiatry with the National Drug Treatment Centre, Dublin, Ireland. **Ciara O'Connor** is a registrar in psychiatry at St Patrick's University Hospital, Dublin, Ireland. **Anne M. Doherty** is a consultant liaison psychiatrist at Mater Misericordiae University Hospital, Dublin, and an Associate Professor of Psychiatry at University College Dublin, Dublin, Ireland.
