# Supplementary material for: Research needs of higher specialist trainees in psychiatry in Ireland: mixed methods study
Source: BJPsych Bull. 2025 Oct;49(5):342–7. doi: 10.1192/bjb.2024.91 (PMC12501522; doi:10.1192/bjb.2024.91)
Supplement: Counihan et al. supplementary material 1 — Counihan et al. supplementary material [file S2056469424000913sup001.pdf]

A pilot study to determine the research educational needs for Higher Specialist Trainees in Psychiatry in Ireland

Research is a key component of evidence-based medicine, clinical medical education, and is also a requirement for all trainees of the College of Psychiatrists of Ireland (CPsychI) at both basic (BST) and higher (HST) specialist training. Previous studies have identified a lack of familiarity in research methods and perceived lack of supervision as barriers to participation in research.

The aim of this study is to explore the needs of HST trainees, and to develop resources to facilitate trainee research participation and encourage a culture of research.

* Indicates required question

1. Please confirm that you have read and understood the **Information Leaflet** about this research project. *

   Mark only one oval.

   - Yes
   - No

2. Please confirm that you understand that you do not have to take part in this study and that you can opt out at any time. *

   Mark only one oval.

   - Yes
   - No

3. Please confirm that you consent to take part in this research study. *

   Mark only one oval.

   - Yes
   - No

Survey

4. What year of the HST programme are you? *

Mark only one oval.

- 1
- 2
- 3
- 4
- 5
- 6
- 7
- Other: ____________________

5. Are you on the Child & Adolescent or Adult Training Scheme? *

Mark only one oval.

- Child & Adolescent
- Adult
- Other: ____________________

6. If you are dual training, are you training in

Mark only one oval.

- Psychiatry of Old Age
- Learning Disability
- Other: ____________________

7. Do you have a clear plan for your research day? *

Mark only one oval.

- Yes
- No

8. Do you have adequate access to research supervision? *

Mark only one oval.

- Yes
- No

9. If you have adequate access to research supervision, who is providing this supervision?

Mark only one oval.

- Educational Supervisor
- Other: ______________________

10. If you do not feel you have adequate research supervision, please outline what you feel you need:

____

____

____

____

____

11. If you have engaged in research over the course of the HST, have you continued with one research project with one HST mentor or multiple projects and mentors? *

Mark only one oval.

- One
- Multiple

12. Have you any suggestions on how the value of the Research Day might be improved for you? *

13. Would you value the opportunity to use this time for another purpose? *

14. Are there any specific research-related learning outcomes you feel are currently unmet? *

15. Are you aware of a trainee mentoring service for research? *

Mark only one oval.

- ( ) Yes
- ( ) Vaguely
- ( ) No

16. If there was a Research Mentorship Programme in the College, would you be interested in availing of it? *

Mark only one oval.

- ( ) Yes
- ( ) No

17. Why or why not? *

____

____

____

____

____

18. What would you like such a service to look like? *

____

____

____

____

____

19. Where do you primarily find yourself becoming stuck with research? *

Tick all that apply.

- [ ] Forming a research question
- [ ] Literature Review
- [ ] Methodology
- [ ] Finding a supervisor
- [ ] Ethical Approval
- [ ] Sourcing materials
- [ ] Sampling
- [ ] Data Collection
- [ ] Statistical Analysis
- [ ] Scientific Writing
- [ ] Publication
- [ ] None
- [ ] Other: ______________

20. As a HST trainee, would you be interested in supervising a BST conducting research? *

Mark only one oval.

- ( ) Yes
- ( ) No

21. If you answered yes to the previous question, at what stages would you be happy to supervise such research?

Tick all that apply.

- [ ] Forming a research question
- [ ] Literature Review
- [ ] Methodology
- [ ] Ethical Approval
- [ ] Sourcing Materials
- [ ] Sampling
- [ ] Data Collection
- [ ] Statistical Analysis
- [ ] Scientific Writing
- [ ] Publication

22. Have you ever:

Tick all that apply.

	Pre-HST	During HST	Other
**Published a paper in print**	☐	☐	☐
**Published a paper online**	☐	☐	☐
**Presented a poster**	☐	☐	☐
**Given an oral presentation**	☐	☐	☐

23. Have you already started or completed a research degree?

Tick all that apply.

	Started	Completed
**Yes; Masters**	☐	☐
**Yes; PhD**	☐	☐
**Yes; MD**	☐	☐
**No**	☐	☐

24. Do you have further comments/questions that are not covered by the questions above? *

This content is neither created nor endorsed by Google.

Google Forms
