# Supplementary material for: Research needs of higher specialist trainees in psychiatry in Ireland: mixed methods study
Source: BJPsych Bull. 2025 Oct;49(5):342–7. doi: 10.1192/bjb.2024.91 (PMC12501522; doi:10.1192/bjb.2024.91)
Supplement: Counihan et al. supplementary material 2 — Counihan et al. supplementary material [file S2056469424000913sup002.pdf]

Theme	Subthemes	Supporting quotes
Collaborative research culture	Isolation<br><br><br><br>Motivation<br><br>Peer support	*'Part of research is being able to collaborate and network with the experts, you need to know who to talk to and be proactive in your approach, projects that are imposed on uninterested people tend to have low value'.*<br><br>*'I need someone to motivate me and prompt me'*<br><br>*'Advice from people who have done similar research to myself, at a peer to peer level'*<br><br>*'I have very limited experience of research and would be helpful to join other research projects with trainees to see a completed research project prior to starting your own'*
Choice	Choice of project that trainee is interested in<br><br><br><br>Choice of supervisor<br><br>Choice of how to spend time – several participants said they would rather use the time for other things	*'advertisement of research projects so that trainees can get involved with projects of interest'*<br><br>*'project or supervisor with similar research interests'*<br><br><br>*'prefer to choose who my research supervisor or mentor is'*<br><br>*'If it helps career development- though don't plan on being involved in academic(s)'*<br><br>*'psychotherapy training'*<br><br>*'undertake diploma, masters unrelated to research'*<br><br>*'the time should also be available for post graduate education'*<br><br>*'allowing additional qualification e.g. psychotherapy'*<br><br>*'can the focus move from research to more opportunity to train up in management , healthcare systems , leadership, psychotherapy etc '*<br><br>*'choosing to do clinical work'*<br><br>*'training in psychotherapy would be much more useful. Or training in management and leadership'*<br><br>*'the grid. There's no other time to do all the outcomes on it'*<br><br>*'educational activities'*
Guidance	Supervisor factors	*'Educational supervisor has reluctantly agreed to supervise in absence of another consultant'.*

	Planning<br><br>Experience/lack of experience	*'Struggled to find a mentor. Not something that was of interest to a lot of consultant(s) where I am working. Those that were are very selective of who they help with research.'*<br><br>*'Now that I have a research supervisor I feel that I am learning and proceeding with research; earlier in my career it would have been helpful to have more guidance around getting involved in research and planning projects.'*<br><br>*'research takes years of planning - its not something you can just conjure up at the start of your HST'*<br><br>*'Clearer guidance in early stages of HST re building research question, progressing ethics and accessing research supervisor - ideally within first few weeks of commencing HST each July, to then use as guidance for rest of training. At later stages of training this could focus on academic writing and getting published, or building grant applications etc.'*<br><br>*'A better system to allow trainees to engage in research of interest across HST rather than ad hoc projects.'*<br><br>*'Academics who are publishing are time poor and have multiple trainees and/or limited areas of interest and therefore fostering and developing research skills are restrained.'*
Access to resources	Time as a resource<br><br>Finance	*'clinical responsibilities can bleed into this time'*<br><br>*'boundaries between clinical and non-clinical work'*<br><br>*'The grid. There's no other time to do all the outcomes on it'.*<br><br>*'Postgrad education, such as MD/PhD, might not be financially feasible for some, even with TSS grant etc. '*

	Geographic constraints<br><br>Access to Journals, librarian support, statistician support	*'It would be interesting if there were a central hub (like the jobs advertised on the college's website) where consultant researchers could list projects they need help with, and trainees could contact them.'*
